# Bioenergetics and the Evolution of Cellular Traits

**DOI:** 10.1146/annurev-biophys-070524-090334

**Published:** 2025-05

**Authors:** Paul E. Schavemaker, Michael Lynch

**Affiliations:** Biodesign Center for Mechanisms of Evolution, Arizona State University, Tempe, Arizona, USA

**Keywords:** cell biology, energetics, evolution, fitness

## Abstract

Evolutionary processes have transformed simple cellular life into a great diversity of forms, ranging from the ubiquitous eukaryotic cell design to the more specific cellular forms of spirochetes, cyanobacteria, ciliates, heliozoans, amoeba, and many others. The cellular traits that constitute these forms require an evolutionary explanation. Ultimately, the persistence of a cellular trait depends on its net contribution to fitness, a quantitative measure. Independent of any positive effects, a cellular trait exhibits a baseline energetic cost that needs to be accounted for when quantitatively examining its net fitness effect. Here, we explore how the energetic burden introduced by a cellular trait quantitatively affects cellular fitness, describe methods for determining cell energy budgets, summarize the costs of cellular traits across the tree of life, and examine how the fitness impacts of these energetic costs compare to other evolutionary forces and trait benefits.

## 1. INTRODUCTION

From the dawn of life on Earth, evolutionary forces have wrought great transformations in the cellular fabric, resulting in the great diversity of cellular forms around the planet in the present day. Ciliates, amoebozoans, choanoflagellates, heliozoans, and chlorophytes are just a small sample of this diversity within eukaryotes alone. Scenarios have been put forth to explain many cellular traits: eukaryotic flagella (60, 61), ciliature in ciliates (20), endomembranes (6, 8, 16, 17, 27, 28, 34, 35, 59), phagocytosis (11, 12), and the nucleus (36). Most of these scenarios for the evolution of cellular traits lack a quantitative accounting of the net fitness consequences of the traits and their intermediates. These net fitness changes depend not only on the downstream effects on survival and reproduction but also on a baseline fitness effect that derives from the energetic cost of a trait (48, 51).

Cellular traits are burdens as well as boons. Nothing is for free. This is as true for cells as it is in human affairs. Cellular traits are generally composed of proteins, lipids, carbohydrates, and nucleotides. When a new membrane-enveloped compartment, appendage, or metabolic pathway is introduced, resources need to be devoted to the synthesis of the constituent parts. There are two options: (*a*) The resources are added on top of the preexisting cellular budget, extending the time required for the cell to transport and assimilate these resources and prolonging the cell division time. (*b*) The resources are taken away from preexisting traits, negatively affecting survival, growth rate, or both. Under either scenario, when the contribution of a trait to net fitness is evaluated, the resource cost must be accounted for alongside the benefit.

One convenient way of accounting for the resource burden is to define the energetic cost of the trait in units of ATP hydrolyses (48, 52, 98), the primary energy currency of the cell. This brings proteins, lipids, nucleotides, and other organic molecules under the same accounting system and unifies the definitions of construction and operating costs. The construction cost consists of both direct and opportunity costs (54), with direct cost referring to chemical conversion of ATP molecules and opportunity cost referring to the potential energy associated with biomass.

Once an accounting mechanism for trait costs is established, the costs of individual traits can be examined. The composition of protein complexes can be determined by X-ray crystallography or electron microscopy, and by fluorescence microscopy for larger, more loosely organized assemblies. Protein lengths can be obtained from genome sequences and their abundances by proteomics or fluorescence microscopy. Membrane areas can be obtained from electron microscopy. Specific examples are examined in Section 5.

The impact of a trait cost on fitness depends on the total cell energy budget. A trait that costs 0.01% of the cell budget has a different impact on fitness than a trait that costs 10% of the cell budget. The cell budget depends strongly on the cell volume (48). Various methods for estimating the cell budget, with different strengths and weaknesses, are available and are discussed in Section 4. Once both a trait cost and a cell budget are known, a relative cost can be determined. The relative cost defines the negative baseline fitness impact of the newly introduced cellular trait, independent of any downstream benefits.

The fate of a new trait can be understood when the fitness cost, associated with the resource burden, is combined with the fitness benefit that the trait bestows upon the cell. This yields a net fitness change upon introducing or modifying a trait. If this net fitness change is larger than the power of genetic drift, and if the change is not opposed by a strong mutational pressure (45), the trait can sweep through the population under the power of natural selection (47, 52). An exact fitness benefit is difficult to determine given uncertainties about the ecology of single-celled species. Plausible estimates can be arrived at in some cases, and these are discussed and compared to the fitness impacts of the energetic costs.

## 2. THE FITNESS IMPACT OF A CELLULAR TRAIT

Evolutionary change occurs gradually, often one mutation and fixation event after another. In time, this leads to the development of distinct cellular traits. For both the (immediate) small steps and the (longer term) distinct trait differences, it is important to understand the fitness impact of a change between an ancestral state and a derived state. A new trait comes with both positive and negative impacts on fitness, and the combination of these effects determines the fate of the cellular modification. Negative impacts can be chemical cross-reactivity (23), chaperone burden (40), surface area occupation (87, 88, 94), or blocking of transport in the cytoplasm (68), but here we primarily examine the resource burden in terms of the energetic costs of construction and operation. In this scenario, the fitness of a derived state is given by (51)

1. $$\text{Fitness}=1-C_{\text{trait}}+s_{\text{benefit}}.$$

This fitness is a function of the fitness of the ancestral state normalized to 1, the relative energetic cost of the trait as a fraction of the cell budget, $C_{\text{trait}}$, and the selection coefficient of the downstream effect of this trait on fitness, $s_{\text{benefit}}$. If the absolute value of this fitness differential exceeds the power of genetic drift, the trait can be advanced or purged by natural selection (47).

## 3. THE FITNESS IMPACT OF RESOURCE COMPETITION BETWEEN CELLULAR TRAITS

The impact of the energetic cost of a cellular trait on fitness, in Equation 1, is linearly proportional to the relative cost. This was surmised by Orgel & Crick (70), used by Lynch & Marinov (48) and Wagner (98) to understand gene evolution, and put on a more solid footing by Ilker & Hinczewski (32) (see also 51). Ideally, the impact should be tested experimentally by introducing nonfunctional proteins and examining the effect on cell fitness. An attempt at such an analysis involved a cost-benefit analysis of the *lac* operon (18; for earlier work, see 41). In this experiment, the *lac* operon expression level was altered artificially in the absence of lactose, such that the cell experienced the burden of expressing the operon but did not benefit from metabolizing lactose. Examination of the growth rate at different *lac* operon expression levels revealed a decrease in growth rate larger than that predicted by a linear model (18). The growth defect may have resulted in part from an increased chaperone burden or cross-reactivity of *lac* operon proteins with other cellular traits rather than entirely from the direct consequence of an energetic burden.

**Figure 1:**
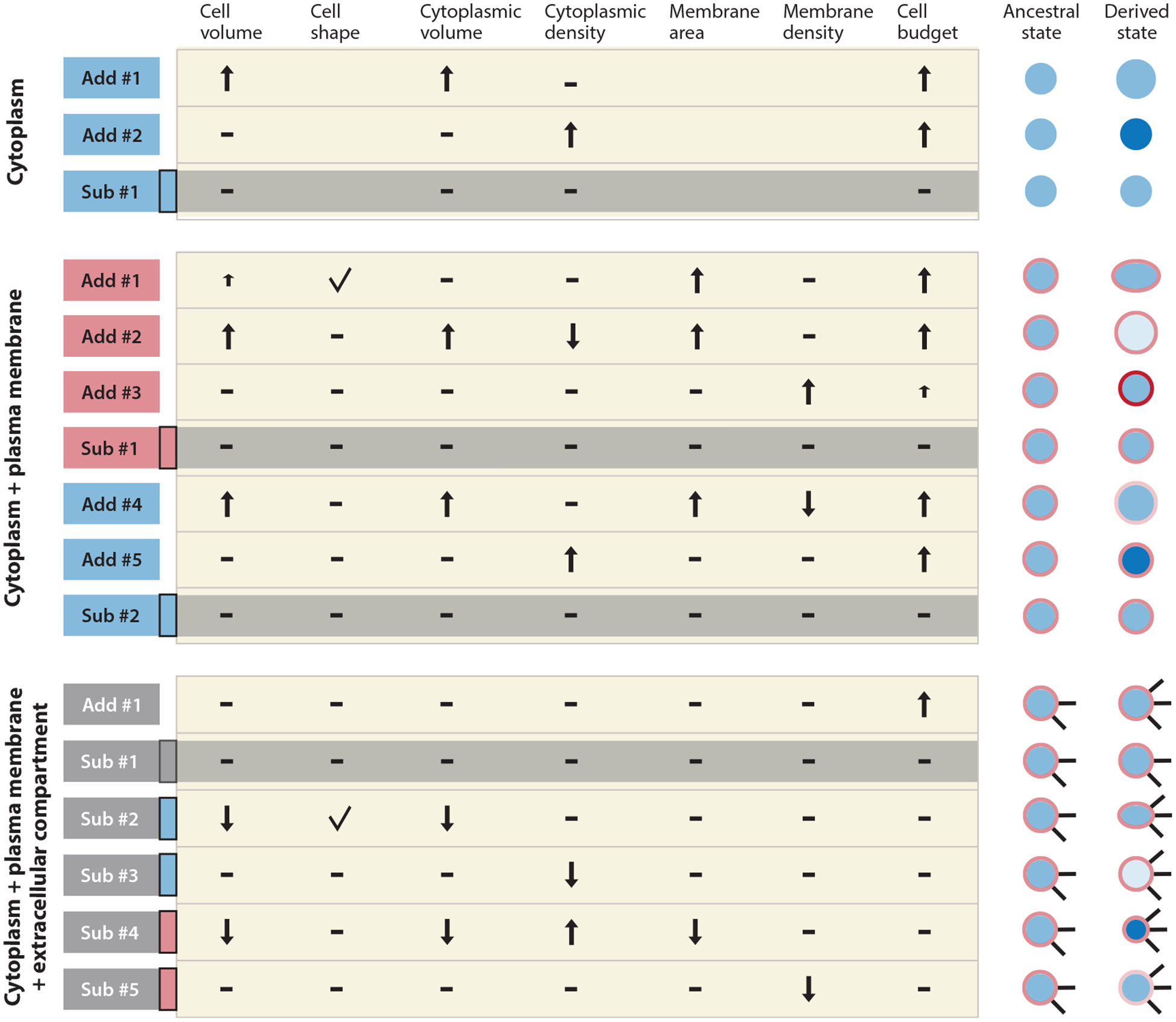
Consequences of adding or substituting a cellular trait. Examining three abstract cell types: only cytoplasm, cytoplasm + plasma membrane, and cytoplasm + plasma membrane + extracellular compartment. New cellular traits are added to, or substituted into, the cytoplasm, the plasma membrane, or the extracellular compartment—indicated by the colors blue, red, or dark gray, respectively. For substitutions, the compartment from which the resources are removed is indicated by the color in the black-rimmed boxes. Upward and downward arrows indicate increase and decrease of change, small arrows indicate small changes, check marks indicate that a change has happened in cell shape, and dashes indicate no change. The diagrams on the right show the cells before (ancestral state) and after (derived state) the introduction of a new cellular trait. Blue is the cytoplasm, red is the plasma membrane, and black is the extracellular compartment. Changes in the density of the cytoplasm or plasma membrane are shown as changes in color intensity. The lightgray-shaded areas highlight substitutions for which none of the parameters listed at the top change. Abbreviations: Add, addition; Sub, substitution.

The impact of the energetic cost of a cellular trait on fitness is crucial for the comparison with trait benefits, and it is therefore worthwhile to explore this matter more thoroughly, especially in the context of the cell. The introduction of a new trait into a cell can happen by addition or replacement, and it can be put in the cytoplasm, the plasma membrane, or the external medium (Figure 1). Here, additions to and replacements of organelle membranes and lumens are treated as an addition to the cytoplasm but these may require separate treatment in the future. How and where a trait is introduced have consequences for the accounting of resource costs. For conceptual clarity, first let us consider a cell that consists of only cytoplasm and no plasma membrane or external proteins. Introducing a new trait, say, a set of enzymes that constitutes a metabolic pathway, can then have one of three effects (Figure 1): (*a*) The cell grows in volume (37), (*b*) the cell increases its density, or (*c*) the resource investment in preexisting traits is reduced. These effects can occur simultaneously.

The first two of these effects increase the overall cell budget and therefore the assimilation requirement for completion of the cell cycle. If the assimilation machinery is saturated and does not change in abundance, the increased cell budget causes a delay in the completion of the cell cycle and thereby contributes negatively to fitness (32, 48, 51). The delay in the completion of the cell cycle is due to a change in the ratio between, for example, ribosome abundance and protein production requirement. Such ratio effects are more general and exist for chaperones, protein-modifying enzymes, metabolic pathways for amino acids, and the production of osmoprotectants, among others. An increase in volume may also have ecological fitness-associated effects. An increase in density may slow down diffusion, causing an additional reduction in growth rate.

The third effect has simpler consequences for the cell. It lacks fitness consequences for changes in cell volume and cytoplasmic density, but it suffers from the same ratio effect that plagues the other two options. The preexisting traits lose investment and therefore lose proteins (and other components), reducing the ratio of the protein abundance of each preexisting trait to that of the entire cell.

Next, consider a cell composed of a cytoplasm enveloped by a plasma membrane (Figure 1). As above, a new cellular trait can be added, increasing the cell budget, or it can substitute (parts of) other traits, keeping the cell budget constant. The addition or substitution can happen in either the cytoplasm or the plasma membrane. The addition of a plasma membrane–localized trait would change the cell shape, increase the cell volume, or increase membrane density—depending on how the trait was added. These changes affect fitness independent of the increase in the cell budget. The substitution of preexisting plasma membrane–localized traits would cause no such baseline fitness impacts and keep the cell budget constant (Figure 1). Adding a new cellular trait to the cytoplasm increases the cytoplasmic density or requires a larger investment in the cell membrane. Again, the substitution of traits keeps all of these parameters and the cell budget constant.

Finally, consider a cell that has a cytoplasm, a plasma membrane, and external features such as appendages or excreted proteins (Figure 1). Adding a trait externally increases the cell budget but does not affect any of the other cellular parameters considered above. A substitution of (parts of) external traits with a new external trait has no effect on any of the discussed cellular parameters. Substitution of cytoplasm- and plasma membrane–localized traits with externally localized traits has no effect on the cell budget but does affect cell volume, membrane area, or compartment density, all of which may have effects on fitness unrelated to energetic costs (Figure 1).

For analytical simplicity, it is best to consider substitution scenarios, as the effects are confined to the diminishment of the fitness of the preexisting traits. Trait-addition scenarios typically feature additional fitness effects, except for the addition of an external trait (e.g., a flagellum). However, whether a new cellular trait is added to the cell budget or substituted depends on the behavior of actual cells and may differ between traits or species. It may also depend on the timescale considered. If a new trait is the consequence of a single mutation, the choice between addition or substitution depends on the regulatory system that is already in place. If the new trait emerges from an accumulation of many mutations over a long period of time, there is an opportunity to reorganize the cell by regulatory mutations and the cell will be optimized with respect to the new trait. This may involve a choice between addition or substitution as well.

Energetic costs of cellular traits have been discussed in relation to an energy limitation (1, 51, 53). However, a unicellular organism has a particular cell volume required by its ecological niche, and a particular macromolecular density because this optimizes transport rates. These factors, independently of any energy limitation, set the cell budget and the impact of the energetic cost of cellular traits on fitness. In this view, the energy limitation does not determine the cell energy budget but affects the cell division time.

## 4. ESTIMATING CELL ENERGY BUDGETS

The evolutionary impact of the energetic cost of a trait depends on the relative cost of this trait with respect to the cell budget. Methods such as measuring nutrient uptake rate, thermodynamic calculations, knowledge of metabolic pathways, and metabolic models have estimated aspects of cell budgets for various species (5, 22, 25, 56, 62, 66, 71, 93, 97, 100, 101). Some studies have focused on just the metabolic rate (19, 21, 24, 65), extreme energy limitation (31), how proteome cost is minimized (39), or how eukaryogenesis has affected the energy budget (15, 43, 48, 49, 63, 64). Here, we discuss four methods for estimating the energy budget of a cell: from the Pirt plot approach using chemostat data, from the heat of combustion and elemental composition of cells, from metabolic rate estimates, and from macromolecule volume fraction and membrane surface area. Each of these methods has advantages and limitations.

**Figure 2:**
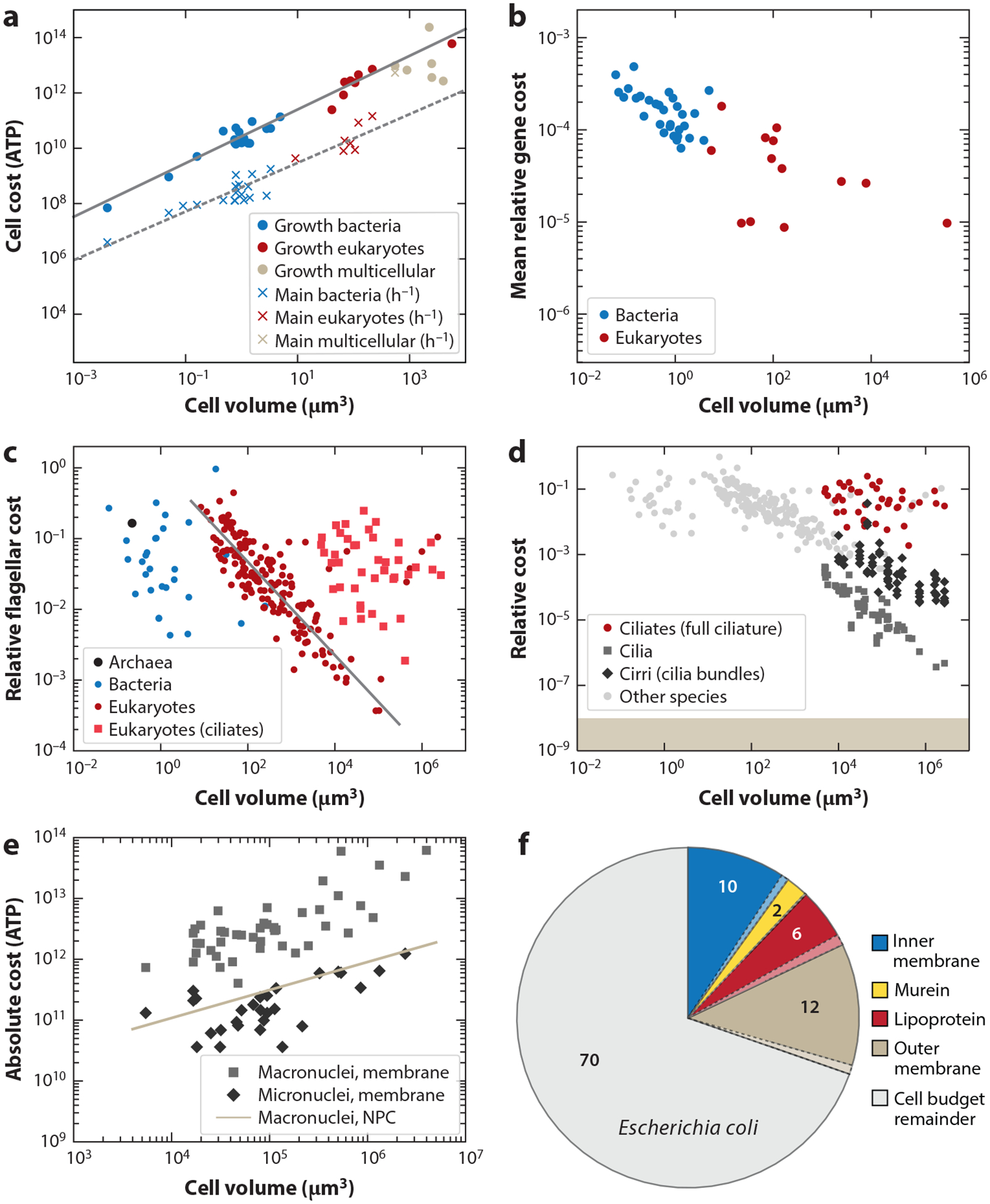
Cell budgets and the energetic costs of cellular traits. (*a*) Cell budgets of various species of bacteria and unicellular and multicellular eukaryotes. Cell budgets are subdivided into a growth component and a maintenance component (48). The regression lines are $27{V_{\text{cell}}}^{0.97}$ (*solid line*) and $0.39{V_{\text{cell}}}^{0.88}$ (*dashed line*) in units of 10^9^ ATP. (*b*) The mean gene cost, relative to the cell budget, for various species of bacteria and unicellular eukaryotes (48). (*c*) Flagellar construction cost, relative to the cell budget, for archaea, bacteria, and eukaryotes (50, 86). The dashed line is given by $0.98{V_{\text{cell}}}^{-0.66}$, which is a fit to the eukaryotic flagellates (excluding the hyperflagellated eukaryotes, mostly ciliates). (*d*) Cost of individual cilia or cirri (cilia bundles), relative to the cell budget, compared to the cost of the full ciliature of ciliates (50, 86). The shaded region indicates the cost of a cellular feature that selection cannot remove, assuming that all ciliates share the effective population sizes (*N*_e_) of the ciliate genera *Tetrahymena* and *Paramecium* (51). The costs for archaea, bacteria, and the remaining eukaryotes from panel *c* are included for comparison. (*e*) Absolute cost of macronuclear and micronuclear membranes for various species of ciliates. The cost of nuclear pore complexes (NPCs) is indicated by the line, $C_{\text{NPC}}=1.57\times 10^{9}{V_{\text{cell}}}^{0.46}$. This equation is obtained by combining a power law that relates macronuclear volume to ciliate cell volume (50) with a power law that relates the NPC energetic cost to the nuclear volume. (*f*) Cell envelope costs in *Escherichia coli* compared to the cell budget (51). Numbers show percentages. Panels *a* and *b* adapted from Reference 48. Panels *c* and *d* adapted from Reference 86 (CC BY 4.0). Panel *f* created using data from Reference 51.

In the Pirt plot approach (48, 50, 73), cells are grown on glucose (or other simple substrates) in a chemostat at different dilution rates. The rate of consumption of the limiting substrate (e.g., glucose) is measured at the different dilution rates (equivalent to growth rates), and knowledge of metabolic pathways is then used to convert the glucose consumption into units of ATP, the primary energy currency of the cell. The implications of different energy metabolisms, for example, respiration versus fermentation, on the calculation of the energy budget require further examination. The conversion of glucose into units of ATP allows for the determination of a cell duplication (growth) cost and a maintenance cost, with the growth costs being incurred just once per cell cycle and the maintenance cost increasing linearly with the cell cycle time. This method requires that cells can be grown in a chemostat on a simple energy source. When cells are consuming other cells, the conversion of the organic matter to ATP equivalents becomes prohibitively complex. Fortunately, prior results from applying this approach to a diverse collection of species have been used to derive the relation between the energy budget of a cell and the cell volume (48) (Figure 2a),

2. $$C_{\text{cell}}=27{V_{\text{cell}}}^{0.97}+0.39t_{d}{V_{\text{cell}}}^{0.88}.$$

Here, the cell energy budget $C_{\text{cell}}$ is in units of 10^9^ ATP, $t_{d}$ is the cell division time in hours, and $V_{\text{cell}}$ is the cell volume in cubic microns.

The second method for estimating cell energy budgets uses the elemental composition of cells. A measure called the degree of reduction can be calculated from the number of carbon, hydrogen, and oxygen atoms in a chemical compound (50). A variant of the degree of reduction also includes phosphorus atoms (52). This degree of reduction correlates strongly with the heat of combustion and the ATP requirement for synthesizing those compounds (52). Using the cell dry weight, the mass fraction of carbon in that dry weight, and estimates of the C:H:O ratio allowed for the determination of a scaling law for ciliates (single-celled eukaryotes) that relates the cell volume to the cell budget in units of ATP (50),

3. $$C_{\text{cell}}=52{V_{\text{cell}}}^{0.92},$$

where, as above, $C_{\text{cell}}$ is in units of 10^9^ ATP and $V_{\text{cell}}$ is in cubic microns. Using elemental data for two bacterial species and two yeast (including phosphorus) yields a cell budget estimate of 4.7 × 10^10^ ATP for a 1 μm^3^ cell, compared to 2.7 × 10^10^ ATP from Equation 2, when excluding the maintenance cost (52).

A third estimate, also derived for ciliates, uses cell growth rates and oxygen consumption rates (metabolic rates). These are combined with an estimate of the ratio of energy in carbon skeletons to the metabolic rate to arrive at an estimate of (50)

4. $$C_{\text{cell}}=115{V_{\text{cell}}}^{0.77},$$

with units as above. It is assumed that in these ciliates, energy metabolism is limited mostly to oxidative phosphorylation.

**Figure 3:**
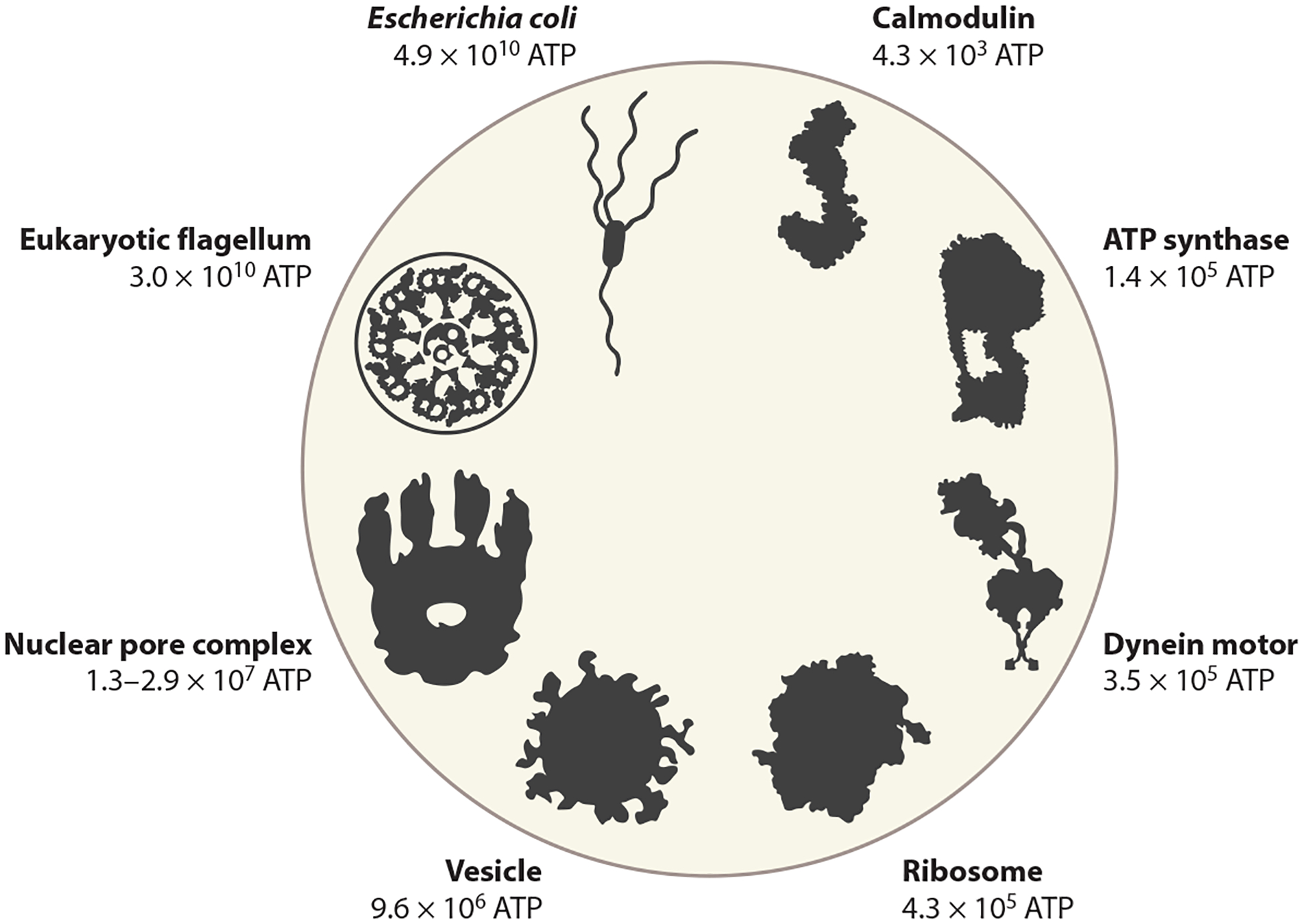
The energetic cost of cellular traits. Costs were calculated for individual proteins, for macromolecular complexes, or, in the case of *Escherichia coli*, for a single cell (shown for comparison). Calmodulin: 148 amino acids; ATP synthase: 4,955 amino acids (*E. coli*); dynein-2 motor: 12,084 amino acids (human, PDB ID: 6SC2); ribosome: 7,459 amino acids and 4,566 ribonucleotides (*E. coli*); vesicle: diameter 50 nm [membrane cost including proteins = 1.45 × 10^9^ ATP μm^–2^ (86)]; nuclear pore complexes: 50–110 MDa (yeast and vertebrates) (2, 7); eukaryotic flagellum: 11 μm in length; and *E. coli*: estimated using the fourth cell budget method. Abbreviation: PDB ID, Protein Data Bank identifier.

A fourth estimate of the cell budget derives from knowing what compartments constitute a cell and how many lipids, proteins (57, 58), and other constituents are in each compartment. This approach has been used to estimate the cost of introducing a vacuole into a cell in which cytoplasm, plasma, membrane, vacuole membrane, and cell wall are treated as separate compartments (79). Consider a bacterium, modeled after *Escherichia coli*, with five compartments: cytoplasm, inner membrane, periplasm, outer membrane, and external filaments. The areas and volumes of the first four compartments can be obtained from micrographs. Using a cell volume of 1 μm^3^, a periplasm width of 0.02 μm, and a macromolecule volume fraction of 0.16 (13, 14, 42) and including four flagella as external filaments (86), we calculate the total cell cost as 4.85 × 10^10^ ATP (Figure 3). Here, it is assumed that all macromolecules in the cytoplasm and periplasm are proteins. Protein costs (48) and membrane costs (49, 86) are derived from knowledge of biosynthetic pathways of amino acids and lipids. This cell budget estimate is in reasonable agreement with that of the other methods, but because it accounts for specific features associated with the cell of interest and not the average features of a diverse set of organisms, we should not expect exact agreement.

There are two advantages to this fourth method. First, it can account for species-specific differences in cytoplasmic density and abundance of external biomass, which the regression-based methods average out. Second, cytoplasmic density (91) and abundance of external biomass (e.g., filaments and excreted proteins) can be tuned in an evolutionary model and optimized together with other traits of interest that make a demand on cellular resources.

### 4.1. A Cell Budget Is Not Fixed over Evolutionary Time

Over evolutionary time, cells can grow or shrink in volume, increase or decrease in density, modify the number of external appendages, or adjust the amount of excreted biomass—and thereby change the cell budget. They can also crank the operating cost up or down (e.g., by swimming more or less vigorously). In addition to this, the flux of nutrients into the cell, be it for energy or specific chemical elements, can change and shift the resource limitations. This can happen by a change in cell shape, nutrient transporters, swimming speed, or hunting behavior. The impact of these cell budget changes on cellular evolution remains largely unexamined.

### 4.2. Alternative Resource Accounting Methods

Energy is not the only resource on which the cell depends, and it is worthwhile to consider the impact of resources. Cost-benefit analyses have been performed with respect to chemical elements such as carbon, nitrogen, and phosphorus (79, 81, 83). An explicit calculation of the fitness consequences of an elemental resource burden has not been carried out but would presumably work in the same way as for energy, except that it cannot account for maintenance cost. How fitness is affected when accounting for multiple elements and energy at the same time has yet to be examined.

When cellular parts contain the same energy density [i.e., the same amount of proteins and lipids (which differ somewhat in energy density) per unit volume], a volume ratio can be used as a proxy for cost. This has been used to determine costs of dinoflagellate flagella (83) and proto-mitochondrial symbionts (88). In a similar vein, one could examine the impact on another limiting resource, the cell surface area. This has been used to explain why bacteria enter overflow metabolism at fast growth (94, 102). It was also used, together with energetic costs, to calculate the fitness effects of primitive vesicle systems that occupy the plasma membrane during their construction (87).

## 5. THE ENERGETIC COSTS OF CELLULAR TRAITS

The energetic costs of cellular traits are central to determining the net fitness consequences of introducing these traits. Here, we start with a summary of the energetic costs for various traits and then explain the importance of these costs in relation to effective population sizes (*N*_e_), trait benefits, and physical models of the evolution of cellular traits. This discussion focuses on the construction costs of cellular traits. Operating costs can be a significant contributor to trait costs in some cases (86).

Understanding the cost of a cellular trait involves three questions (78): (*a*) How fast does it perform its task? (*b*) How big is it? (*c*) How many are there? Here, “it” is any unit that performs a task. This unit could be an individual metabolic enzyme, catalyzing a single reaction—but it could also be a motor protein, a nuclear pore complex (NPC), an endoplasmic reticulum to Golgi complex transfer vesicle, or a mitotic spindle.

The introduction of a trait burdens the cell by way of energy (48, 79, 82), elemental requirement (e.g., C, N, or P) (79, 81, 83), biomass (77), volume (83, 88), or a combination thereof. Here, we focus on energetic costs, expressed in units of ATP hydrolyses, which include both direct costs and opportunity costs (52, 54), defined in Section 1. The other methods of accounting for the cellular burden of a trait are mentioned when necessary. To make trait costs evolutionarily relevant, they must be converted into relative costs by normalizing the cell budget. Energetic costs are often reported as watts or joules, but for comparison these are converted into units of ATP, assuming 55 kJ mol^–1^ ATP (58), in the following examples.

Most cellular traits are products of genes, so the cost of individual genes is a good starting point for an enumeration of cellular trait costs. Gene costs are associated with replication, transcription, and translation, and cost estimates for the full gene complement of a diverse set of organisms reveal that the average relative energy cost of individual genes decreases with cell volume and that translation costs are higher than the costs for transcription and replication (48). Average gene costs, relative to the cell budget, are shown for bacteria and eukaryotes in Figure 2b. Wagner (98), by a similar method, estimated that the relative cost of duplicating the median gene is 4.68 × 10^–5^ in *Saccharomyces cerevisiae*, which (given the cell volume) is consistent with the relative costs in Figure 2b.

Energetic costs of individual macromolecules can be computed when the monomer composition is known (1, 3, 48, 72, 74, 75, 90, 98). As examples, the energetic costs of the small protein calmodulin, the protein complexes ATP synthase and dynein-2, and the protein- and RNA-containing ribosome are shown in Figure 3. Cellular trait costs are sums of the costs of gene products, including, but not limited to, proteins, membranes, and cell wall components. Some cellular traits are composed of only proteins (e.g., the circadian clock, gas vesicles, and the cytoskeleton). The energetic costs of these (and other) traits are listed in Table 1.

Cellular traits such as vesicles, organelles, and (some) cellular appendages contain membranes, and an overview of membrane-associated costs is shown in Table 1 and Figure 3. For the membrane costs of mitochondria in ciliates, a power law relation has been estimated: $2.3\times 10^{9}{V_{\text{cell}}}^{0.98}$ ATP (50). If the exact composition of an organelle is not known, volume fractions, of the organelle compared to the volume of the whole cell, can be used as a (rough) proxy for cost. This, for example, allows for a comparison of mitochondrial and chloroplast costs, with mitochondria at volume fractions ranging from 0.46% to 15% (88) being less costly to cells than chloroplasts with volume fractions frequently between 30% and 40% (78, 96). These ranges reflect natural variation, not measurement error.

Flagella are ubiquitous cellular appendages used for swimming and their cost can be estimated from structural studies of the protein components and (when present) from the membrane area (51, 83) (Figure 3; Table 1). A survey of 200 species of bacteria and single-celled eukaryotes showed that in most cases the relative construction cost of flagella is between 0.1% and 40% (86) (Figure 2c). The relative costs of individual cilia (flagella) and cirri (cilia bundles) for many species of ciliates are shown in Figure 2d. These costs reveal what benefit the addition of a single cilium or cirrus must bring to the cell before the net fitness change is positive. They also reveal that the addition of even a single cilium or cirrus can be detected by natural selection (by comparison with the inverse of the effective population size, 1/*N*_e_, see below) (Figure 2d).

The nucleus is a defining characteristic of eukaryotic cells, and the energetic costs of the envelope can be evaluated by examining its membranes and pore complexes. Any benefits proposed for the evolution of the nucleus (11, 36) need to exceed these costs for an evolutionary scenario to be plausible. Nuclear membrane cost can be estimated from nuclear surface area. For ciliates, this yields an energy cost of $6.8\times 10^{9}{V_{\text{cell}}}^{0.54}$ ATP (macronucleus) and $1.5\times 10^{9}{V_{\text{cell}}}^{0.41}$ ATP (micronucleus) (50, 86) (Figure 2e). For other species, including phototrophs and mammals, the scaling is similar: $7.8\times 10^{9}{V_{\text{cell}}}^{0.48}$ (52, 86). Using the nuclear volume as a proxy for the relative cost of the whole nucleus (including, e.g., the genomic content), the relative nuclear energetic cost for ciliates is given by $2.82{V_{\text{cell}}}^{-0.41}$ (macronucleus) and $0.16{V_{\text{cell}}}^{-0.54}$ (micronucleus) (50). For a typical ciliate cell volume of 10^5^ μm^3^ this yields relative costs of 2.5% (macronucleus) and 0.032% (micronucleus). The nuclear volume fraction can be higher in multicellular organisms, with angiosperm cells devoting 20% of their volume to the nucleus (58). Algae have nuclear volume fractions between 4.4% and 20% (78). Nuclei are studded with NPCs that regulate the transport between the nuclear lumen and the cytoplasm. An individual yeast NPC costs 1.3 × 10^7^ ATP, and for vertebrates this is 2.9 × 10^7^ ATP, using respective molecular weights of 50 Mda (2) and 110 MDa (7) (Figure 3). When an average of these values is used, the cost of all NPCs combined scales with the nuclear volume as $7.0\times 10^{8}{V_{\text{nuc}}}^{0.78}$ ATP (52).

Energetic costs have also been studied for trichocyst discharge (Table 1), compatible solute synthesis and ion import in response to osmotic stress (69), vesicle release and action potentials (4), membrane protein insertion (30), membrane curvature generation (92), cellular sensing (29), cell cycle oscillator (85), noise regulation by microRNAs (33), and silicon use (76).

### 5.1. Energetic Costs of Cellular Modifications and Genetic Drift

Natural selection is not all powerful. It can be counteracted by mutation pressure and genetic drift (46, 47). An extra base pair in a gene, an extra amino acid in a protein, and an increase in expression level all come with energetic costs that are detrimental to cell growth and survival, unless these changes come with additional positive effects on cellular functioning. Even in the absence of such positive effects, the modifications can still be fixed in the population if the energetic cost of an addition is small enough compared to mutation pressure or genetic drift. Gene frequencies in finite populations are subject to random fluctuations (genetic drift) with magnitudes proportional to *N*_e_. *N*_e_ values are generally far smaller than those of census population sizes and depend on genetic phenomena such as recombination and background mutation rates as well as on demographic factors. Fitness effects smaller than (roughly) 1/*N*_e_ are invisible to selection (47). To determine the fate of a cellular adjustment, be it the accidental duplication of a gene or a single amino acid insertion, it is therefore important to compare the fitness effects to 1/*N*_e_.

**Figure 4:**
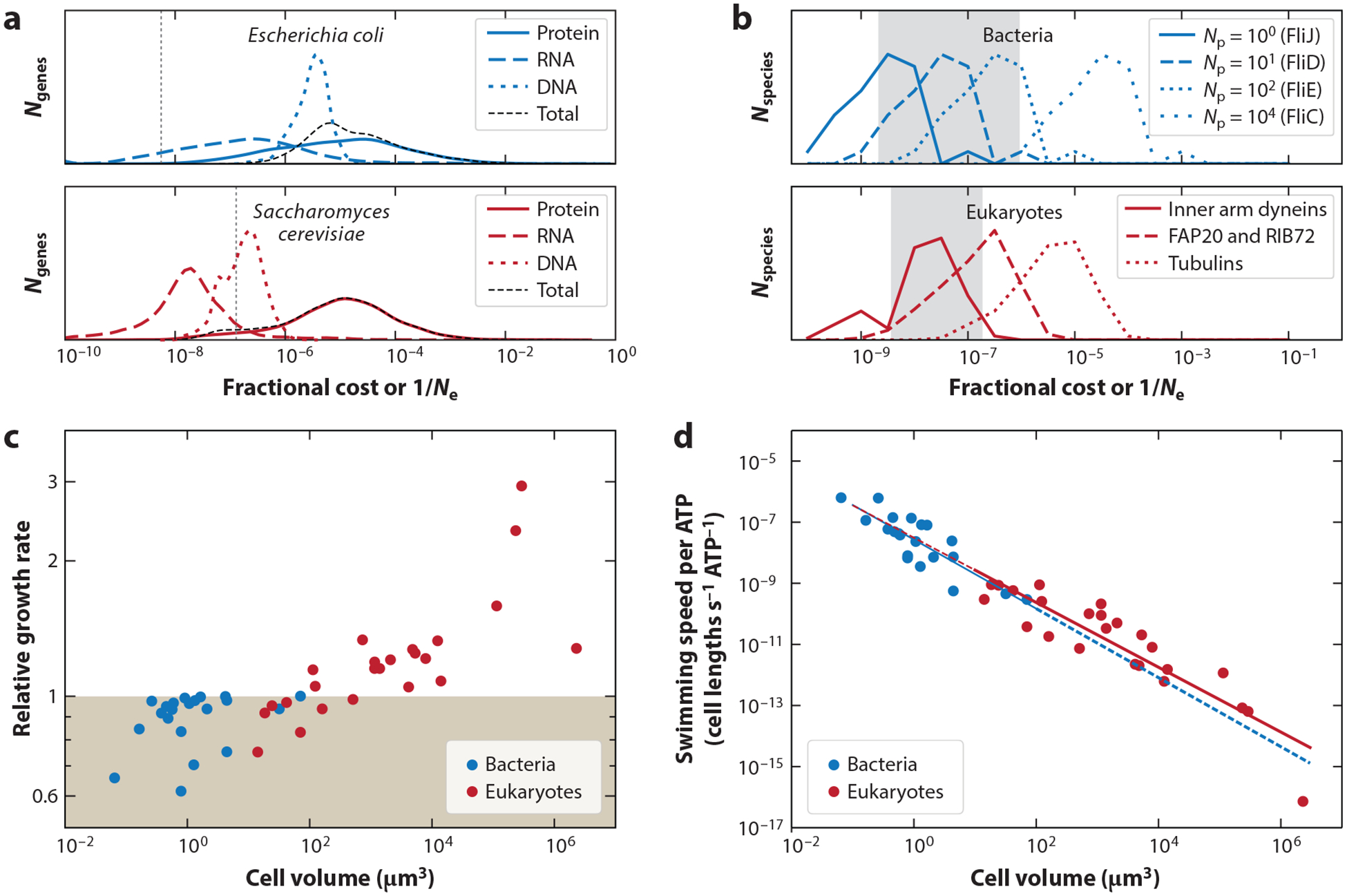
Energetic costs compared to the power of selection and trait benefits. (*a*) Fractional cost of genes, compared to the cell budget, in *Escherichia coli* (bacterium) and *Saccharomyces cerevisiae* (eukaryote) (48). *N*_genes_ is the number of genes. Vertical dotted lines show the inverse of the effective population sizes (1/*N*_e_) for *E. coli* and *S. cerevisiae* (51). If the addition of a gene has a fractional cost smaller than 1/*N*_e_, it is effectively invisible to selection (48). (*b*) Fractional cost of adding a single (average) amino acid to different flagellar proteins in various species of bacteria and eukaryotes (86). *N*_species_ is the number of species and *N*_p_ is the (approximate) number of proteins of a particular type in a single flagellum. The gray-shaded regions indicate the range of 1/*N*_e_ for diverse bacteria and unicellular eukaryotes (51). If an amino acid addition has a fractional cost smaller than 1/*N*_e_, it is effectively invisible to selection (86). (*c*) Cost-benefit analysis for organisms swimming in a homogeneous, as opposed to patchy, medium of small-molecule nutrients (86). Relative growth rate shows the balance between the costs of adding a flagellum and the gain in nutrients from swimming. The shaded region indicates where the growth rate is reduced in the presence of a flagellum. (*d*) Swimming speed per ATP invested in the construction of flagella for bacteria and eukaryotes (86). The solid blue line is a fit to the bacterial data, $2.7\times 10^{-8}{V_{\text{cell}}}^{-1.13}$, and the solid red line is a fit to the eukaryotic data, $3.1\times 10^{-8}{V_{\text{cell}}}^{-1.06}$. The dashed lines are extrapolations. Panel *a* adapted from Reference 48. Panels *b*–*d* adapted from Reference 86 (CC BY 4.0).

The energetic cost of the duplication of any gene in the *S. cerevisiae* genome, and its accompanying doubling of the expression level, has been estimated (98), showing that for all genes a duplication event is costly enough to be detected by natural selection. In the same organism, an expression-level change of >10% for mRNA and >2% for protein, for a gene with median expression level, would be visible to natural selection; below these thresholds changes could occur neutrally (98). Gene costs in *E. coli* conform to the conclusion that gene duplications are visible to selection, but gene costs in *Caenorhabditis elegans* and *Arabidopsis thaliana* are low enough, compared to the effects of genetic drift, that many genes can be duplicated without opposition from natural selection (48) (Figure 4a). Single amino acid additions to proteins in eukaryotic and bacterial flagella can be visible or invisible to natural selection depending on the copy number of the protein within the flagellum and cell (86) (Figure 4b). A comparison of energetic costs of DNA and *N*_e_ in viruses reveals that small stretches of DNA can persist in larger viruses even in the absence of a selective benefit (53).

### 5.2. Comparing Costs and Benefits

Traits do not just burden the cell. They also endow it with benefits. It is the balance between costs and benefits that determines the net fitness effect—and that, together with genetic drift and mutation pressure, decides the fate of a trait and its elaborations. In many cases, it is difficult to quantify the fitness effects of trait variants, as the benefits often depend on unknown aspects of the external environment, but analyses of costs and benefits have been performed for vacuoles, osmoprotection, flagella, vesicles, and other traits, which we discuss in the following paragraphs.

Vacuoles in photosynthetic cells can be used to increase the average photon capture efficiency of chromophores by spreading these chromophores over a larger area with respect to the incident sunlight. This is the result of a central vacuole turning the cell into a shell of cytoplasm surrounded by a cell wall. At a fixed cytoplasmic volume (excluding the vacuole) the addition of a vacuole increases plasma membrane and cell wall costs and introduces vacuolar membrane costs and costs of maintaining vacuole osmotic balance. Comparing a spherical 5-μm-radius nonvacuolate cell to a spherical 11.1-μm-radius vacuolate cell with the same cytoplasmic volume shows that there is a 2.1-fold increase in cellular cost, from 4.2 × 10^13^ to 8.7 × 10^13^ ATP (mostly cell wall) (79). The benefit of vacuolation, by increased photon uptake, can exceed the cost if the chromophore concentration is higher than some threshold value (79; see also 80).

Cells in freshwater face a constant influx of water by osmosis, a problem that can be solved either by synthesizing a rigid cell wall or by constantly expelling water by contractile vacuoles. For a spherical algal cell with a radius of 5 μm and a cell division time of 24 h, the energetic cost of a cell wall is 2.75 × 10^7^ ATP s^–1^, and the cost of operating a contractile vacuole is 3.78 × 10^7^ ATP s^–1^, the cell wall being increasingly favored as the cell grows more slowly (75). The cost of the contractile vacuole is so high that there is a substantial selective pressure on keeping the cytoplasmic osmolarity low in organisms with lifestyles that exclude a cell wall, with large consequences for the operation of other cellular traits (75). In ciliates, the construction cost of a contractile vacuole is 1.8 × 10^5^ ATP s^–1^ (assuming a cell division time of 24 h), which is only a minor contribution compared to the operation cost mentioned above (50). Cell envelope costs for bacteria have also been estimated (51, 95), typically making a major contribution to the cell budget (Figure 2f).

The flagellar construction cost in terms of both energy and nitrogen composition has been examined in a model dinoflagellate (83). In a scenario of this dinoflagellate swimming up in the water column to increase its light-harvesting capacity and down to increase its nitrogen uptake, the flagellum pays back for itself many times over (83). A similar analysis can be performed for cells of different volumes and swimming speeds that live in a homogeneous distribution of small-molecule nutrients. Only large cells can improve their nutrient uptake beyond the investment in flagella (86) (Figure 4c). A cost-benefit analysis can also be used to compare the capacities of the bacterial and eukaryotic flagella, which function by wildly different mechanisms. Plotting the swimming speed per ATP invested in flagella against cell volume reveals that the bacterial and eukaryotic flagella follow a common trend. They appear to be equally effective at generating swimming speed, though they function at different cell volume ranges (86) (Figure 4d). The cost-benefit relation for chemotaxis has also been examined (67).

Other cost-benefit analyses are of photon absorption (26, 77), active water transport and osmotic compound synthesis for the purpose of buoyancy (44, 82), the cell volume gain accomplished by taking up a proto-mitochondrial respiring symbiont (88), gene regulation in a variable environment (18, 38), and responses to high photon flux (84).

### 5.3. Trait Models for Cost-Benefit Analyses

The costs and benefits discussed so far consist of systems as they exist in present-day species. For understanding evolutionary transitions or for understanding why seemingly plausible alternatives do not exist, it is important to perform cost-benefit analyses on cellular traits that deviate from what presently exists. Such analyses require models in which the investment in a trait can be varied and in which the benefit to the cell can be computed from this investment.

In the study of early endomembrane evolution, direct examples of intermediate states are lacking and need to be constructed from plausible subcellular parts. One example of such a cellular part is the vesicle, which has been extensively studied in contemporary species (e.g., 89). Vesicle properties, such as size, plasma membrane area occupancy, construction cost, and construction time, can be combined in a model with functional elements that reside on those vesicles. Placing nutrient transporters on those vesicles yields a primitive form of pinocytosis, while adding Sec translocases facilitates membrane protein insertion and yields a proto-endoplasmic reticulum. Cost-benefit analyses can be performed by varying the abundance (investment) of those vesicles, revealing that pinocytosis of small-molecule nutrients does not improve fitness under sensible parameter ranges but that the proto-endoplasmic reticulum does improve fitness (87).

## 6. CONCLUDING REMARKS

Cellular traits exhibit both fitness costs and benefits, with their balance determining the likelihood of establishment and continued existence of a trait in a population. The relative energetic cost of a cellular trait confers an inescapable baseline fitness penalty. An absolute energetic cost of a cellular trait is assembled by combining the known costs of basic building blocks such as amino acids, lipids, sugars, and nucleotides. This is converted into a relative energy cost by dividing it by the cell energy budget. Absolute costs have been estimated for a circadian clock, cell walls, flagella, vacuoles, osmoprotection, mitochondrial and nuclear membranes, and NPC, among others. Cell budgets have been estimated for species across the tree of life. Combining the absolute trait costs and the cell budgets helps reveal what evolutionary scenarios are plausible and what cellular configurations are possible.

## Figures and Tables

**Table 1:** Costs of cellular traits

Cellular trait	Energetic cost	Comments (references)
Circadian clock	2 × 10^8^ ATP^a^ 5 × 10^5^ ATP (operation) 1.5%^b^	Composed of the proteins (in *Synechococcus*): KaiA, KaiB, and KaiC (52)
Cortical microtubules	5.0 × 10^10^ ATP	Idealized *Ochromonas* cell (9, 10,75)
Cytoskeletal filaments (actin and tubulin)	0.1%, 1.4 × 10^9^ ATP (*Saccharomyces cerevisiae*) 0.4%, 1.1 × 10^10^ ATP (*Schizosaccharomyces pombe*) 5.7% (mouse fibroblasts) 0.6% (HeLa cells)	Total amount in the cell (52)
Cytoskeletal filaments (MreB and FtsZ)	0.2% (*Escherichia coli*)	Total amount in the cell; MreB is an actin homolog, FtsZ is a tubulin homolog (52)
Endoplasmic reticulum + Golgi complex	1.1% (*Ostreococcus tauri*) 1.1% (*S. cerevisiae*) 3.0% (*Dunaliella salina*) 21% (*Sus scrofa*, pancreas cell)	Membrane costs only (49)
Flagella	2.3 × 10^8^ ATP (*E. coli*) 3.0 × 10^10^ ATP (*Chlamydomonas*) 4 × 10^11^ – 3 × 10^12^ ATP (cirri)	Cirri are flagellar bundles in ciliates (50, 86)
Gas vesicles	3.1 × 10^9^ ATP	For keeping an *E. coli*-sized cell buoyant (99)
Membrane	1.25 × 10^9^ ATP μm^−2^ (pure lipid) 1.45 × 10^9^ ATP μm^−2^ (40% protein by area)	49, 86
Mitochondrial membrane	1.9% (*Dunaliella salina*) 4–17% (ciliates) 6.7% (*S. scrofa*, pancreas cell) 7.9% (*Ostreococcus tauri*)	Inner and outer mitochondrial membrane combined (49, 50)
Trichocyst discharge	1.4 × 10^7^ ATP	Cost for discharge over the entire cell; trichocysts are defensive structure in ciliates (55)
Vesicle	1.67 × 10^7^ ATP (during construction) 9.6 × 10^6^ ATP (cytoplasmic)	Vesicle diameter: 50 nm. Cost during construction is an average over the 140-s construction period and includes the cost of actin, clathrin, and other proteins (86, 87)

a Costs expressed in ATP are construction costs, the amount of energy it takes to build the cellular trait from scratch, unless indicated otherwise.

b Costs expressed as percentages are relative to the cell budget.

## FUTURE ISSUES

1. The exact quantitative relation between the relative energetic cost of a trait and its fitness consequences is unknown. This should be studied in various evolutionarily relevant scenarios, in diverse cellular configurations, and for different growth models.
2. Various methods for determining cellular energy budgets and trait costs yield similar results. However, there are inconsistencies between some studies, and these require further study.
3. Cellular traits do not appear in a full-fledged, all-or-none fashion; they are advanced incrementally. This has implications for the analysis of costs and benefits and requires knowledge of mutational step sizes at a phenotypic level.
4. A wealth of data on cellular ultrastructure, trait dynamics, and macromolecular composition are now available. This knowledge allows for the determination of the energetic costs of many more traits, which can then be used to evaluate the plausibility of evolutionary scenarios.
5. For the evaluation of alternative evolutionary scenarios, it will be important to establish physical models of cellular traits in which both the baseline fitness penalty and the benefit are a function of the relative energetic cost.

## Abbreviations

Cellular trait: any feature of a cell that affects reproduction, survival, or both; typically under the influence of genes
Fitness: relative reproductive and/or survival advantage of an individual; generally compared to the mean in the population
ATP: an organic molecule used as the primary energy currency in the cell to drive protein-catalyzed reactions out of equilibrium
Opportunity cost: cost of using a nutrient as a building block in macromolecules; equivalent in ATP number to burning it for energy
Genetic drift: changes in the frequency of a variant in a population due to chance
Chemical cross-reactivity: a chemical compound reacting in a process unrelated to its purpose, with potential detrimental effects on fitness
Chaperone burden: misfolded proteins can be refolded by chaperones; introducing labile proteins requires an increase in chaperone abundance, burdening the cell
Selection coefficient: increment of advantage or disadvantage of a derived state compared to an ancestral state; compare with fitness
Eukaryogenesis: the evolutionary transition that led to large and complex cells with nuclei, mitochondria, endomembranes, and a cytoskeleton
Overflow metabolism: switch of energy metabolism from respiration to fermentation at fast growth; observed in *Escherichia coli* and *Saccharomyces cerevisiae* (yeast)
Effective population size (*N*_e_): population size relevant for determining the consequences of, e.g., genetic drift; typically smaller than the census population size
Mutation pressure: statistical directionality of mutations toward or away from a specific set of states; explains some features of genome composition
